# A realist synthesis of cross-border patient movement from low and middle income countries to similar or higher income countries

**DOI:** 10.1186/s12992-017-0287-8

**Published:** 2017-08-29

**Authors:** Jo Durham, Sarah J. Blondell

**Affiliations:** The University of Queensland, School of Public Health, Herston, Qld 4006 Australia

**Keywords:** Patient mobility, Cross-border medical travel, Capital

## Abstract

Patient travel across borders to access healthcare is becoming increasingly common and widespread. Patients moving from high income to middle income countries for healthcare is well documented, with patients seeking treatments that are cheaper or more readily available than at home. Less well understood is when patients move from one low income country to another or from a low income country to a higher income country. In this paper, a realist review was undertaken to explore why, in what contexts and how patients from lower income countries travel to countries with the same, or more advanced, economies for planned healthcare. Based on an initial scoping of the literature and discussions with key informants, we generated an initial theory and set of propositions about why, how, who and in what contexts people cross international borders for planned healthcare. We then systematically located and synthesized (1) peer-reviewed studies from the Scopus, Embase, Web of Science and Econlit databases; (2) non-indexed reports using key informants and Google; and (3) papers from the reference lists of included documents, to glean supportive or contradictory evidence for our initial propositions. As we reviewed the literature and extracted our data, we drew on the work of Pierre Bourdieu to understand the interplay between material and non-material capital and cognitive processes in decisions to cross borders for healthcare. Patient travel was largely undertaken due to a lack of services in the home country and/or unacceptability of local services, with decisions on when, and where, to travel, usually made within the patient’s social networks. They were able to travel via use of multiple resources, including social networks, economic and cultural capital, and habitus. Those patients with greater volumes of the aforementioned factors had greater healthcare options; however, even those with limited resources engaged in patient travel. Patient movement challenges traditional ways of thinking about public health and the notion of health systems contained within the nation state. Further research is needed to better understand the effects of patient travel, and how to harness the benefits of patient travel without exacerbating existing health inequalities.

**Electronic supplementary material:**

The online version of this article (doi:10.1186/s12992-017-0287-8) contains supplementary material, which is available to authorized users.

## Background

The World Health Organization (WHO) defines health systems as ‘all the activities whose primary purpose is to promote, restore or maintain health’ [1]. This definition recognises that the health system extends beyond the public health sector. Most analyses of the health system, however, are limited to an examination of the public sector contained within geo-political territorial boundaries [2]. Increased integration into global and regional markets and the commodification of health is, however, contributing to increased patient mobility, as patients cross international borders in search of health services and products [3–7]. Restricting health system analysis to national public sector services, whilst attractive in terms of planning, distorts our understanding of the health system, provides a misrepresentation of health-seeking practices, and masks the true burden of disease [8]. A better understanding of ‘real world’ patterns of healthcare engagement and patient mobility, therefore, is important in ensuring optimal health outcomes for people in both the sending and receiving country, and in beginning to explore new ways of managing health systems that extend across borders [9].

Travel for healthcare is not a new phenomenon [9, 10]. Within the last decade, however, the global trade in health services and products has rapidly expanded, with some governments explicitly including this trade in health in their national economic development plans [10, 11]. Much patient movement involves travel from the Global North to the Global South in search of treatments that are cheaper and more affordable than at home, or where waiting lists are shorter, and this has been the focus of most scholarly research [11–14]. Less attention has been paid, however, to the South-South flow of patients, despite the recognition that much international medical travel comprises South-South or South-North flows [4, 10, 15, 16]. The primary objective is to address the questions of: why do patients from low and middle income countries (LMICs) cross international borders for healthcare?; what are the contextual factors which influence this choice?; how do patients cross international borders?; and who crosses international borders for planned healthcare? To our knowledge, no previous realist synthesis has examined South–South or South-North medical travel.

## Methods

While there is no set definition for what constitutes a realist review, other than it is underpinned by realist logic and its constructs, the RAMESES project has produced training materials and reporting guidelines which were used to inform and guide our synthesis at the different stages of the review [17]. As the study did not involve primary research, it did not require formal ethical approval. The study did, however, follow the ethical standards of utility, usefulness, feasibility, propriety, accuracy and accountability [18].

### Changes to the review process

The initial intent of the review was to follow a rapid realist review approach [19]; however, this was subsequently revised. This decision was informed in discussions with other researchers who have undertaken realist reviews. In particular, we wanted to produce explanations that were potentially transferrable across contexts and populations, rather than the very context-specific explanations typically generated in a rapid realist review [19]. We also felt that as we were not evaluating a specific intervention with a clearly articulated program theory, engaging in theory at the scoping stage was important [20]. We, therefore, decided to follow the five stages recommended by Pawson in conducting realist reviews [20]. It is important to note that while presented below as five steps, in practice, the review was iterative with the researchers moving between the different steps [21, 22].

### Rationale for a realist synthesis

Realist review is a theoretically-driven, qualitative approach to synthesizing qualitative, quantitative and mixed-methods research evidence [20, 22–26]. It differs from other empirically focused qualitative or quantitative methods in that it is a theory-driven, abductive approach that aims to understand context, mechanism, and outcome (CMO) configurations [25, 27]. We chose a realist synthesis because of the substantial heterogeneity that exists in transnational health systems and the complex interactions that occur within the system. Complex systems consist of multiple human components (clinicians, nurses, allied healthcare staff, patients, carers and so forth) that interact in a non-linear fashion to produce outcomes which are highly context dependent [23, 28–32]. In complex systems, outcomes depend on humans making decisions in a semi-predictable (demi-regular) manner about how to use the resources available to them in particular contexts, making a realist approach more appropriate than a systematic review [22, 27, 33].

Central to a realist review is the generative explanation for causation - that is, outcomes (O) of interest are generated by relevant mechanism(s) (M) being triggered within certain contexts (C). Mechanism refers to a generative process that creates or constitutes a regularity that explains how outcomes follow from decision-making processes and the capacity (resources) to enact these decisions [27]. In the case of cross-border health-seeking practices, it refers to the interaction between the reasoning of patients and their capacity to seek healthcare outside of the domestic market. These mechanisms are influenced by the context. Context, in a realist review, generally refers to aspects of the background (in this case, the health system or health market), people and the research setting, i.e. the location of the study, that moderate outcomes. Context may allow or, alternatively, constrain agency [19, 34]. Together, context and mechanisms interact to bring about outcomes. Outcomes refer to expected or unexpected intermediate (mediating) and final outcomes or results. The C-M-O configurations help ensure external validity, as they allow the research to extend to a level of abstraction for the theory, or theories, to be useful in other contexts [34].

### Scoping the literature

#### Step 1: identifying potential theories

A realist review differs from empirically focused qualitative or quantitative methods in various ways, including its theory-driven and abductive approach to understanding C-M-O configurations. Thus, central to any realist synthesis is developing and refining candidate theories that relate to the area of investigation [27, 33]. Theory is typically incorporated at the beginning of the review and informs the development of the protocol [20, 27].

At the start of the current study, the initial review completed as part of the study protocol was further refined through further reading of how health markets are expected work [35]. In this initial scoping of the literature, our focus was on understanding how healthcare service markets (as a sub-sector of health systems markets) were expected to work through the process of demand and supply, rather than specifically focussing on patient mobility. In addition to the literature, we drew on face-to-face discussion between ourselves and colleagues who are involved in global health system research, as well as on our experiential knowledge, observation and our familiarity with the access to health services research literature. This understanding was then used to construct the theoretical framework [33], which underwent multiple iterations, both prior to starting the review and as our understanding grew through our research.

We recognised that healthcare markets, however, typically do not function in the way of ideal markets, as described in neo-classical economics [36–42]. Thus, from the outset, we theorised markets, and healthcare markets in particular, as interactive socioeconomic institutions that work through process of interactions of demand and supply [36, 40, 43], with patient consumers and healthcare providers participating in a social contact with healthcare goods or services exchanged within a system of social relations and networks [44, 45].

We hypothesised that people value health and, as such, are willing to invest in the commodity of ‘good health’ in a variety of ways, including through the purchasing of health services and products. In our initial review, we identified numerous demand- and supply- side factors that may lead to market failure. Demand-side determinants influence the ability to use health services, and include household resources and willingness to pay, perception of illness, self-care practices, information on healthcare options, distance from health services, opportunity costs, cultural and social factors, prior experiences and subjective assessment of quality of services [46–48]. Supply-side determinants include technological and diagnostic capacity, costs of services, availability and quality of staff and responsiveness to patient demand [40, 46, 49, 50]. These demand- and supply-side features are not necessarily mutually exclusive, and may interact and influence each other. They are often grouped under four facets of access: availability, geographic accessibility, affordability and acceptability [46, 47, 51–53]. When applied to people from lower income countries crossing borders for healthcare, we hypothesised that when domestic markets fail, due to an interaction between the supply- and demand-side factors of availability, geographic accessibility, affordability and acceptability (context), and where patients have the resources to access information on available healthcare services and cross borders (context – possibility to choose), some patients would be willing to invest in the commodity (good health) by crossing-borders (market mechanism). We assumed that the patient-consumer’s therapeutic destination would be one that was perceived as accessible, affordable and with acceptable levels of service, as well as offering the required services. The definitions we used in this review to describe these supply- and demand-side determinants of access are outlined below in Table 1.

**Table 1:** Definitions of supply- and demand-side determinants

Availability	Availability of the right kind of care to those who need it, such as hours of operation and waiting times (S) that meet demands of Patients (D), and appropriate type of service providers and materials (S)
Geographic accessibility	The physical distance or travel time from service delivery point to the user. Depends on location of patient (D) and location of health services (S)
Affordability	Relationship between price of services (S) and willingness and capacity of users to pay for those services (D)
Acceptability	Match between responsiveness of healthcare services to the social and cultural expectations of individual users and communities

*S* supply, *D* demand

### Searching process

#### Step 2: search strategy

To map the elements of healthcare service markets, with a view to further uncovering the underlying C-M-O configurations that helped explain why patients from LMICs cross borders, we conducted a search of the literature. Searches were conducted to identify studies across the databases Scopus, Embase, Web of Science and Econlit, using the search terms (‘medical tour*’ OR ‘health tour*’ OR ‘patient mobility’ OR ‘medical mobility’) OR ‘therapeutic itinerar*’ OR ‘medical travel*’ OR ‘international patient’ OR ‘transnational health care’ OR ‘transnational healthcare’ OR ‘cross-border health care’ OR ‘cross-border healthcare’ OR ‘cross-border care’ OR ‘health seeking behaviour’ OR ‘patient movement’) AND (health care system’ OR ‘healthcare system’ OR ‘health services market’ OR availability OR accessibility OR affordability OR adequacy OR acceptability OR satisfaction) AND (patient OR tourist OR client) AND (‘low income’ OR ‘middle income’ OR ‘low-and middle-income country’ OR ‘South – South’ OR ‘global south’ OR ‘intra-regional’). The search was further limited to studies published between 2000 and 2014, in the English language. This was due to budgetary constraints, and as the authors speak only English.

Additionally, multiple papers for possible inclusion where known to the authors, based on their previous work on the topic, and these papers were also screened. Given that grey literature is a relevant source of information for realist reviews, a Google search was also undertaken to identify other materials including, for example, reports published by governments, international organisations, non-governmental organisations, dissertations and theses [20, 22]. A further less exhaustive database search was also conducted to update the results. This secondary search used less search terms and only one database. Bibliographic references from the included documents were also reviewed, using the snowballing technique, to identify additional documents.

### Selection and appraisal of documents

#### Step 3: study selection criteria and procedures

Titles and abstracts were screened by SJB. To be included in the review, a study was required to meet all of the following criteria, namely (1) To relate to patient travel across borders for access to healthcare; (2) To relate to travel from low to low or low to higher income countries (according to the World Bank classifications [54]); (3) To present quantitative, qualitative, mixed methods data or a review of the literature; (4) To be published between 2000 and 2014; and (5) To be published in English. One investigator (SJB) reviewed the documents and, where unsure of acceptability, a second investigator (JD) was consulted. Each paper was examined for evidence based on how it supported, refuted, reinterpreted or refocussed the novice theories developed, looking at how each study contributed to the initial theory. Relevance and quality were assessed guided by the following questions:

1. Does the paper (or an aspect of it) describe patient movement across borders from a low or lower income country to the same or a higher income country?
2. Does the paper (or an aspect of it) provide information on the context in the sending and/or receiving country?
3. Does the paper (or an aspect of it) provide some evidence that will contribute to the synthesis and our emerging theory?
4. Were the methods used to generate the relevant data credible – e.g. does the research support the conclusions [20, 55]? Is the evidence provided good enough based on sample size, data collection, data analysis, etc. [20, 55]?

Patients were required to intentionally leave their birth country for planned healthcare, with the intention of returning to their birth country. Following Lunt [56], we excluded long-term residents (immigrants) of other countries who use the health services provided by their host country, and patients that received treatment abroad for emergency procedures whilst travelling for a holiday.

### Data extraction

#### Step 4: data extraction

Eligible papers were compiled in an Endnote library, and an accompanying document-summary sheet was developed in Microsoft Excel. The template included factors such as the author name, year, patient sending and receiving country, and study type. In addition, using the theoretical framework to guide themes, an NVIVO coding structure was developed using the definitions provided in Table 1. In practice, however, this proved to be unwieldy to use and a bespoke data extraction form was developed using Excel. Evidence from the documents was initially aligned against the program theory with tentative mechanisms identified using inductive and deductive processes.

### Analysis and synthesis processes

#### Step 5: data synthesis

In synthesising the data, additional codes were created for sections of text that seemed relevant to the program theory. During the coding process, we sought to determine if the coded extract referred to context, mechanism or outcome, what the C-M-O configuration might be and how it contributed to our program theory. We did this through searching for patterns in the data and seeking evidence to support or refute our emerging C-M-O configurations and making comparisons with potential rival theories [27]. From this, we began to further formulate potential C-M-O configurations, which were then tested in the literature. This process enabled immersion in the literature, the search for key terms and testing of hypotheses that could explain for what purpose, in what context, who and how patients crossed borders for healthcare.

From a realist perspective, context, mechanisms and outcomes can work in dynamically changing partnerships [27]. Dissatisfaction with domestic healthcare services, for example, can be an individual contextual factor, or a mechanism, that is, how patients respond to healthcare services, or the result of healthcare services. There is also often a ripple effect, whereby the outcome of one CMO configuration, becomes the context for the next [27]. In this synthesis, while the cultural norms, history, institutional settings and infrastructure of in each paper was different, a common feature was that all patients were travelling from a lower-income county, to a higher income country for healthcare. We defined this as the general context, and looked for individual contextual characteristics [27] as we felt this would more fruitful in identifying more causative CMO configurations. We classified mechanisms as how these individual contextual characteristics interacted with patient responses (for example, cognitive, emotional, and motivational), grouping them thematically. For example, statements about medical travel to save lives for treatment for life-threatening infectious diseases, or where in-country treatments had been futile were grouped under the mechanisms ‘prolonging life’. The result of this interaction, was classified as an outcome. While the outcome that we were looking for was crossing international borders for planned healthcare, as we reviewed our data we noted a ripple effect﻿, whereby interim outcomes became contextual factors for the next CMO configuration. We found it fruitful to record these interim outcomes that contributed to the ‘final’ outcome of interest, that is, crossing international borders for planned healthcare, as a way of producing a more nuanced set of CMO configurations. For example, we found that within a context of patients not being able to obtain the care they needed in-country (context), the mismatch between patients’ perceived needs and services available (mechanism), led to a demand for cross-border healthcare.

As we reviewed the literature and extracted our data, we also noted how supply- and demand-side factors interacted with patients reasoning and their access to resources to follow through to the outcome of crossing the border for healthcare. In this way, we progressively refined our understanding and our conceptualization of the phenomena of cross-border patient travel [57]. Through this process, and informed by our understanding of healthcare markets as interactive socioeconomic institutions, we drew on Pierre Bourdieu [58–61] to understand the interplay between material and non-material capital and cognitive processes in decisions to cross borders for healthcare. Central to Bourdieu’s work are the concepts of economic, social and cultural capital [58]. Table 2 provides an overview of the different types of capital and some of their distinct features. These different types of capital are not independent of each other, with one form of capital being able to be converted to another [58, 62]. Thus, the different capitals cannot be understood in isolation, and need to be examined in relation to how they interact with each other. In Bourdieu’s theory, it is access to these capitals that determine an actor’s freedom of action and his/her chances for profit in a particular social field [58].

**Table 2:** Different types of capital according to Bourdieu [58]

Capital	Definition
Social	“Social capital is the aggregate of the actual or potential resources which are linked to possession of a durable network of more or less institutionalized relationships of mutual acquaintance and recognition—or in other words, to membership in a group—which provides each of its members with the backing of the collectively-owned capital, a ‘credential’ which entitles them to credit, in various senses of the word” [58].
Cultural	Cultural capital is high cultural knowledge that contributes to the owner’s financial and social advantage and is expressed in, for example, style of speech, dress, or physical appearance. It includes health-related values, behavioural norms and health literacy. Cultural capital is also expressed through educational qualifications and through objective, materially represented cultural capital, for example, books, laboratories, medical equipment and scientific instruments.
Economic	Different means of production and other forms of income, such as wages.

Also central to Bourdieu’s work is the concept of habitus. Bourdieu [59] describes habitus as “a system of durable, transposable dispositions, structured structures predisposed to function as structuring structures … as principles which generate and organize practices.” Habitus links objective social conditions to people’s behaviours and often finds its expression in particular lifestyles, including health-seeking practices and, as such, is the central concept at the interface between the individual and the socially structured environment [63, 64]. That is, the concept of habitus helps explain the regularities and patterns of social life, including health-seeking practices, while leaving open the possibility of free and purposeful action [65]. The third important concept in Bourdieu’s work is that of field. A field is a social space of positions and position taking which, while they do not operate as conscious constructions with explicit rules, are, nevertheless, characterized by regularity and norms, with actors investing in the field and struggling for reward as in a game [59]. In Bourdieu’s terms, a healthcare market can be seen as a field, where different types of healthcare workers and institutions coexist and within which they position themselves in order to meet their needs.

We hypothesised that cross-border patient movement is generated by the interaction of valuation processes (related to demand-side contextual factors of availability, geographic accessibility, affordability and acceptability) and the presence of cross-border competition that better meets patients’ needs. We assumed that, where this is the case, patients mobilise their available resources to access this cross-border care. Based on this, we tested three broadly generalizable hypotheses concerning patient movement from lower income countries to higher, or similar, income countries (refer to Table 3).

**Table 3:** Emerging theory of cross-border health-seeking practices

Hypothesis	Anticipated context	Anticipated mechansism	Anticipated outcome
Hypothesis A	Patients perceive a need for healthcare	Mismatch between patient needs (demand), and services (supply) in domestic market	Demand for cross-border healthcare
Hypothesis B	Patients attempt to access cross-border healthcare services due to the mismatch between patient needs (demand), and services (supply) in the domestic market	Patients trust and use social networks and cultural capital to examine and evaluate alternative options	Patient plan to cross border for healthcare
Hypothesis C	Patients plan to cross border for healthcare	Patients mobilise sufficient capital (economic, social, cultural) to cross border for healthcare	Patients with adequate purchasing power (capital), use cross-border healthcare services

### Validity

The iterative process of understanding how healthcare service markets work, and the context in which consumer patients decide to exit the domestic healthcare system for one in another country, required the researchers to move between empirical data and construction of C-M-O configurations [17]. This, alongside the deliberate inclusion of context in the analysis, helped enhance internal validity and the generalisation potential of the identified mechanisms [17, 22].

## Results

**Fig. 1:**
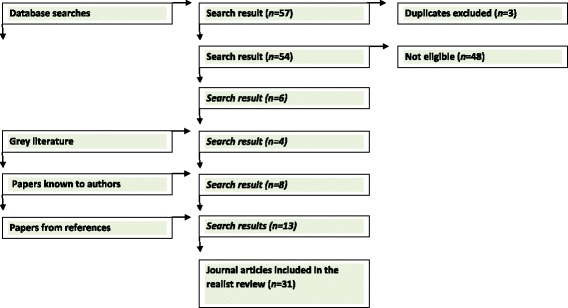
Flow diagram of search strategy

A total of 57 papers were found across the databases, or 54 after duplicates were excluded. Of these papers, six met the inclusion criteria. Additionally, 1 paper was found in the grey literature search and 11 papers were known to the authors to be of interest. From these papers, an additional 13 papers were found from the reference lists that met inclusion criteria. In total, therefore, 31 articles were included in this review. For an overview of the search strategy, please see Fig. 1.

Of the 31 articles retained, papers fell under the broad categories of qualitative (*n* = 13); quantitative (*n* = 9); mixed methods (*n* = 4) and reviews (*n* = 5). Studies were undertaken worldwide, for a breakdown of study sending and receiving countries, in addition to study type, see Additional file 1.

### Why do patients seek healthcare abroad?

Regarding hypothesis A, patients’ or their families’ assessment of the supply–side factors of acceptability, availability and accessibility were key drivers of patient exit from the national health system [2, 10, 11, 15, 66–81]. Based on the review, we identified five principal mechanisms related to the mismatch between demand and supply and one mechanism - gaining citizenship – in which health was not the primary outcome patients desired. These mechanisms are described in brief below. For ease of reference, each mechanism has been given a short explanatory title that captures how the mechanism works. When these mechanisms are triggered, they do not generate health-seeking practices across borders directly; instead, it is the interaction between the mechanism and context that generates the outcome, in this case, crossing international borders for planned healthcare. As shown in Table 3 however, in synthesising the data and developing our emerging theory, we found it useful to develop interim outcomes that then created contexts that contributed to our outcome of interest. In the mechanisms described below, the interaction between the mechanism and the context generated a demand for cross-border healthcare (interim outcome). This formed part of the context for the next CMO configurations, as illustrated in Table 3.

It is important to note that for some people the acknowledgment that the healthcare system in their home country was inadequate, and the subsequent decision to travel abroad for healthcare, was not an easy one; however, one’s health was prioritised over any sense of patriotism. This was expressed by one Yemeni participant in the study by Kangas (76, p. 297):

*‘I want Yemen to learn medicine. I love Yemen. Why would we want a faraway country to treat us for everything? It’s better to spend money inside the country, not outside. However, I do want to live, not die.’*

### Prolonging life!

Travelling abroad in cases where patients or their families perceived life-saving care was needed that was not available, accessible or acceptable locally was common. Even where doctors advised of the futility of travelling aboard, for example, with very late presentations of certain diseases, accepting that there was no hope, especially from medical professionals working in an often distrusted health system, was often seen as not an option [72]. The real or perceived quality of services aboard provided hope, even in severe cases, that a cure might be found. This sentiment was seen across multiple studies, as articulated below:

*“If your country has advanced medicine, you should be treated in your or own country. If it doesn’t exist, then necessity has its rules (75, p. 52)”.*

*“Nothing is free here [in Kuching] but it’s still better than having medical treatment [back home] and not being cured (11, p. 12)”.*

*“Health is the pillar of human life; one has to do anything to obtain it (* [82]*, p.307).”* The outcome of this mechanism (maintaining hope of a cure being found and thus prolonging life) led to a demand for cross-border healthcare. In order to work, however, this mechanism, requires economic capital to cover associated costs, a belief that treatment is available and knowledge of relevant providers across borders (context), with choice of destination country not a random one. Other patients crossed borders to escape life-threatening infectious diseases in their home countries. This mechanism worked in the context of an under resourced domestic health system, combined with an accessible cross-border option (context). For example, when there was a major cholera outbreak in Zimbabwe, during which Zimbabwean hospitals had insufficient drugs, some patients travelled to neighbouring countries, including South Africa, for treatment [15]. While our outcome of interest was patients crossing borders for planned healthcare, we also identified a health system outcome in South Africa, where, afraid of an outbreak amongst its own citizens (mechanism), the government agreed to provide free treatment to all Zimbabweans crossing the border with symptoms (outcome).

### Doing the right thing

This mechanism can operate in conjunction with the prolonging life mechanism just described and also requires knowledge of providers in other countries and economic capital. In people’s social networks, ideas about what was considered good care and the ‘right thing to do’ were constructed as people swapped stories of encounters with the health system at home and abroad. In Kangas’s [72] study, for example, families who had the wherewithal to do so, felt they would experience social criticism if they were not seen to be “doing the right thing” by seeking cross-border healthcare for critically ill family members who could not get the care they needed at home. The “doing the right thing” mechanism, can be considered an anticipatory mechanism, because it operates on the desire to avoid anticipated social censure (negative outcome).

Even though there was less social pressure on those with inadequate resources, many still felt compelled to “do the right thing” and seek services overseas. If the patient did not recover, at the individual or family level another outcome was that families could at least console themselves, that they had done everything they could for their family member.

### Arrogant doctors and distrust

This mechanism may work alone, or in conjunction with the other mechanisms described above, and is based on dissatisfaction with the quality of the clinical encounter, and the extent to which, patients trusted local healthcare providers. In post-conflict Aceh, Indonesia a severe distrust of local health professionals, who were widely accused of being arrogant, incompetent and untrustworthy, led patients to seek healthcare overseas [74]. Descriptions of the Indonesian doctors were in stark contrast to the “friendly” and “intelligent” Malaysian doctors, who were attributed as having a unique capacity to heal. As described in Smith’s study:

*“Over there (Malaysia) the medicine is good, so you can recover quickly. And the doctors are very clever, they’re well educated, and they have good equipment so they only need to make a very small cut. In Aceh, they’d cut you from here to here!* (85, p.11).”

*“Last year my relative had an operation [in East Aceh], and he died during the operation. The doctors here are arrogant and uneducated* (85, p. 283).”

Also in Indonesia, patients from West Kalimantan travelling to Kuching, Malaysia praised the professionalism of Kuching doctors and expressed disdain for the prevalent hierarchical medical culture at home that places patients as passive recipients of care rather than as active consumers [83]. Other common sources of dissatisfaction and mistrust included outdated technology and diagnostic capacity, substandard facilities, uncaring staff and poor quality/counterfeit pharmaceuticals [66, 68, 71–74, 79, 83, 84]. As with the other mechanisms described above, activation of this mechanism requires knowledge of healthcare providers abroad and economic capital. In order for patients to continue using services overseas, they need to feel that the service they receive is of better quality and more trustworthy than that received at home. This involves positive, reinforcing feedback loops, typically, not only at the individual level, but also based on the positive experiences of others in their social networks.

### Fulfilling fertility desires

A particular form of market failure is access to reproductive technologies and human gametes (context). In some countries, lack of access to assistive reproductive technology is due to contextual factors such as the illegality of certain services, or unavailability of services because of real or perceived lack of expertise, equipment, donor gametes or technologies, or services being restricted to people with certain demographic profiles, for example heterosexual, married couples of reproductive age [85–87]. Other contextual factors can include affordability in the home country, or patients concerns about privacy in using domestic services [85]. Activation of this mechanism also requires acceptability to the patient of donated sperm, eggs, and embryos (context).

### Perceptions of faster service and convenience

In most of the sending countries there are referral hospitals, usually in the capital cities, where treatment may be seen as being affordable and of acceptable reasonable quality. Often, however, particularly in border areas, travel to these referral centres necessitates paying for flights, local ground transport, food and accommodation, and means being away from home (context). Particularly in border areas, other contextual factors can include travel across borders being often more convenient, the service superior to locally available services, appointments can often be scheduled in advance and, in many cases, these patients share similar ethno-linguistic traits as in the receiving country, and may have business or familial ties [10, 66, 68, 70, 74, 79]. This is particularly the case where crossing the border is relatively easy and does not necessarily require a visa/passport [15, 71]. This mechanism, therefore, is anticipatory, in that the patient believes that using the cross border services, will save time and reduce the opportunity costs incurred in using local services.

As explained by a participant in the study by Allen [66], when asked whether participants are willing to cross the border from Uganda to Kenya to access healthcare even if it means paying double the rate:

*“Very willing. You are not going to waste time. This is a border town; people are busy doing business. I cannot afford to waste three hours because I’m sitting in a line waiting for service. People are willing to pay whatever amount for their health because they think ‘if I get this service in time, I’ll be able to compensate the money I have used* (67, p. 66).”

### Gaining citizenship

The expectation of potential citizenship for their children sometimes guided the choice of Mexican mothers to give birth in the United States, particularly for women of middle and higher socio-economic status [70]. Contrary to other cross-border patients, however, Mexican patients found the medical staff in the United States impersonal compared to their Mexican counterparts, but valued their technical expertise [70]. In this case, the outcome that patients were seeking was not necessarily better care, but citizenship.

The interim outcome (demand for cross-border healthcare) of these six mechanisms described above, does not explain how the final outcome, that is crossing borders for healthcare, is achieved. Patients also need to determine where to go, how to travel and how to cover the costs. A next step therefore, was to investigate hypothesis B to better understand how patients made these decisions.

### How do patients decide where to travel for healthcare?

Regarding hypothesis B, drawing on the documents reviewed and the work of Bourdieu [58–61], we hypothesised that having decided to seek healthcare overseas patients selected their therapeutic destination based on their experiences and the experiences of those in their social networks. The results revealed that word of mouth recommendations through social networks, including family, friends, business contacts and doctor referrals through their professional networks, were the most reported influence in determining treatment destination [10, 11, 66, 68, 71–73, 81, 85, 88, 89]. For patients, the opinion or recommendations of people they trusted in their social networks and the credibility of these recommendations (mechanism), influenced how patients chose their medical travel destination and provider (interim outcome). Returning travellers often return with medical files and tales of sparkling clean facilities, health professionals who take care of patients and find cures, further enhancing the medical credibility and reputation of therapeutic destinations and providers, or, in Bourdieu’s [58] terms, the symbolic capital of medical care aboard [68, 90]. The powerful role of social interactions was expressed by one participant in the study by Ormond (84, p. 5):

*“Many people suggested that we come to Kuching because they said that the medical treatment here was good. The guy over there [a neighbour who drove her son, sister and husband to Kuching for her husband's treatment] had the experience of falling ill and being cured here [in Kuching]. [ … ] We weren't sure about coming to Kuching, but after we heard about it through word-of-mouth, we decided to come, especially after he [the neighbour] shared his experience of getting sick and being cured here.”*

Within social networks, medical travellers actively articulate what they perceive as the most significant failings of their home health system, and spread information about how to access viable alternatives abroad. Trusted social networks also provided introductions to healthcare providers, assisted with the logistics of medical travel, including providing transport or financial assistance and accompanying patients [10, 11, 68, 89], also assiting in patient destination choice. In these social networks, patients also developed cultural capital by gaining knowledge from others on how to access another healthcare system. While not a focus of our study, another outcome was that in these social networks awareness of chronic diseases, such as cancer, was developed and, patients learned that while often not treatable locally, these diseases could be treated and managed abroad [72, 84].

Certain regions and countries become noted ‘hubs’ for certain ailments and treatments. For Yemeni patients, this manifested in patients seeking eye care travelling to Russia, India for kidney care, Jordan for cancer care. [71]. Likewise, in the Middle East, Iran [87] and Lebanon were hubs for patients seeking assistive reproductive technology, as is the United Arab Emirates [85, 86].

Through these processes patients and family members also experience a sense of solidarity, as they move through their therapeutic journey sharing news and advice on doctors and facilities and details about the medical conditions for which they are being treated [10, 11, 68, 90]. Within social networks, the medical care that family members provide for their relations are also evaluated, shaping ideas about what forms good care. In this way, patients’ dispositions, or habitus and aspirations are also changed as they learn more about the world and the options available to them. This outcome, can have a ripple effect, increasing dissatisfaction with services at home, and an increased preference for services abroad.

While websites, chat rooms, media, specialised agencies and advertisements, access to cell phones and e-mail, that help connect people with social networks beyond their home countries, were also identified as contributing to destination choice, this was to a much lesser extent than word of mouth [68, 85, 90]. Similarly, in some cases, international providers had a commercial presence in a sending country which linked patients to cross-border healthcare [68]. The overall outcome of the interaction between demand for cross-border healthcare and trust in the credibility of the recommendations from trusted social contacts, or through web-based research private providers’ marketing, was that would-be patients started to make concrete plans to travel to a selected treatment destination.

Several of the studies also highlighted the contextual factors of cultural proximity in determining patient destination [10, 68, 71, 91]. The ethno-linguistic similarity between Lao patients along the Lao/Thai border, for example, helped facilitate patient travel [68, 69]. Similarly, patients travelling from Indonesia to Malaysia, from Bangladesh to India, or from Yemen to Jordon shared similar languages and cultures [10, 11, 71, 73]. Zhang and colleagues [92] found that Chinese patients’ subjective perception of the differences in the culture, language, economic and political context between China and potential destination countries was an important contextual consideration in destination choice for relatively minor diseases, but was less of an influencing factor for severer cases. In terms of Bourdieu’s conceptualisation of cultural capital, a level of education and comfort with the foreign health system, as well as travel aids, such as passports, helped shape medical travellers’ treatment and destination options (context).

How do patients travel abroad for healthcare and who are they?

Having decided to travel for healthcare, patients and their families act to mobilise sufficient capital (economic, social, cultural) to put their plans into action. Economic capital is mobilised in different ways. Wealthier patients may have insurance [84] and, in some cases, employers will cover the cost of treatment abroad [72, 77, 90]. In some cases, an important contextual factor was the presence of formal inter-governmental arrangements with and costs covered by the sending country. In South Africa, for example, the government has formal arrangements for intra-regional medical travel with 20 bilateral health agreements with 18 countries in Sub-Saharan Africa agreed [93]. Similarly, patient travel from Libya to Tunisia was facilitated by the Libyan government [94].

In Thailand, an important contextual factor was that based on a decree from the Strategic Office of the Ministry of Public Health, all welfare offices in public hospitals have to treat patients from neighbouring Myanmar, Lao PDR, Cambodia and Malaysia, regardless of their financial means [68]. This guideline is applied both to protect these patient’s right to care, but it also helps to prevent the spread of communicable diseases along the border [68] and in this sense, while not the focus of this review, also represents a health system outcome in response to patient movement. Patient travellers with more access to economic capital, were likely to use private facilities or travel further afield than those with less economic capital [68, 81, 95]. Further, those with cultural capital through education were more likely to have higher levels of economic capital, which in turn, facilitated access to private insurance plans that sometimes included provisions for healthcare across borders providing greater choice in the selection of treatment destinations (context). Many travellers, however, borrowed or sold assets in order to raise sufficient cash to cover the costs of their medical travel and treatment regime in order to pursue healthcare they believed would prolong life [72, 90]. Social networks were also important in helping patients mobilise the necessary financial resources to travel, both prior to leaving and while away [68, 71, 72].

While limited information was available in the reviewed literature on the specific demographic characteristics of these travellers, or the volumes of social, economic and cultural capital they held, they all drew on their different forms of capital to facilitate their medical travel (context). Regardless of the demographic profile, the goal for all medical travellers was to receive appropriate diagnoses and or treatments to promote health. Few of the papers discussed the health outcomes for patient travellers and those that did, did so in general terms. That many patients were satisfied, is seen in how information about the quality and standard of care provided in health facilities are passed on in social networks, adding value in people’s minds to cross-border health-seeking practices and reinforcing a provider’s reputation. On the other hand, for some, the outcome of patient movement included financial debt and disruption in the continuity of care disrupted [95].

## Discussion

This review shows that it is not only patients from industrialized countries who decide to exit their domestic healthcare system, but also patients from LMICs. We identified six mechanisms that interacted with contextual factors to generate a demand for patient travel across borders. While the outcome that we were looking for was crossing international borders for planned healthcare, we found it fruitful to identify interim outcomes that contributed to this “final” outcome. The broad context for each mechanism was market disequilibrium, with unmet need in some lower income countries, but excess supply in other countries of the same or higher income. This mismatch between supply and demand, is partly due to rapid economic, demographic and epidemiological transitions that have given rise to an increase in chronic disease in contexts where health systems are poorly equipped to manage this changing disease burden. Other broad contextual factors include, processes of globalisation and greater integration in regional and global markets. The commodification of health has also facilitated the growth of health insurance companies, and other agencies, that may have a commercial presence in a sending country and link consumers with providers in receiving countries [11].

In order to enact on the desire to seek healthcare across borders, would-be travellers need access to the various capitals identified by Bourdieu [58]. Social capital and trusted social networks, emerged as important in linking patients with medical providers abroad. The study confirms the role of word of mouth, through social networks, as being an important contextual factor in patient destination. Patients often returned from abroad praising the quality of the healthcare received, as well as the good interpersonal relationship developed with healthcare staff. Patients were often loyal to specific specialists, routinely returning for follow-up consultations and recommending them to friends and family and, in this way, specialists gain a good reputation within a patient’s’ social network. Social networks could be used to help solve problems and ‘play the game’ more effectively, and were a potent form of developing cultural capital, as information about services was disseminated through social networks. Through these social networks patients also develop ideas about what health services are like in other places, which in turn and shapes habitus about quality of care, further contributing to market disequilibrium. For people residing in border areas, an important contextual factor was poor physical accessibility to the required health services in the home country, which when combined with the required service being easy to access across the border, triggered the “perceptions of faster service and convenience” mechanism. Often, people in these areas also shared greater cultural ties with the receiving country than the home country, which aided healthcare navigation and use.

While it may be thought that only the wealthy and elite in LMICs would be able to mobilise the resources necessary to access services abroad, this study shows that this is not the case. People of various economic status used a multitude of sources and capitals to facilitate their medical journeys. For those of lower economic means, this form of travel was often funded by numerous sources and, for some families, meant going into sometimes significant debt. In some cases, government schemes were in place to cover a patient’s medical expenses; however, the process (which included written forms) often meant that the most vulnerable were disadvantaged. This highlights the important role of not only economic capital, but also cultural capital. It shows the interplay between cultural and economic capital, and how these can be used to create, or impede, opportunities for better quality care. For patients, with lower access to economic and cultural capital, the most likely mechanisms to be activated are “prolonging life”, “doing the right thing” and if they live close to a border crossing “perceptions of faster service and convenience”. Also of note, is that in most cases several contextual conditions need to be met before a particular mechanism is fired. For example, for the “fulfilling fertility desires” mechanism to fire, there has to be an accessible, acceptable and affordable alternative abroad, with the patient able to mobilise sufficient of the various capitals.

From the above, it is evident that crossing borders for healthcare is not straightforward. Decisions about investments in healthcare services abroad are structured by social networks, the positions individuals hold within these structures, and their access to other capitals. In this way, patients and their families negotiate, in an iterative manner, among competing perceptions and needs, weighing advantages and disadvantages that generate health-seeking practices. Thus, patients’ therapeutic destinations are diverse and influenced by the type of healthcare facilities available, patient capacity to use them, their habitus acquired through social experience, and their holdings of economic and cultural capital [63]. This research provides insights for decision-makers who either want to grow their international market or for those who want to retain their domestic patients. For example, social networks were identified as major sites for sharing information on cross border healthcare options, rather than formal advertising. While availability was often a reason for medical travel, reasons for patient satisfaction (or dissatisfaction) with healthcare services often related to the interpersonal skills of healthcare workers, and was also an important factor in medical travel, highlighting the importance patients place on the relational nature of healthcare.

Unlike many realist synthesis our review was not based on an intervention or family of interventions, for example, programmes that use incentives to change behaviour. In this case, our ‘programme theory’ was elicited from social theory and specifically, how markets trigger certain mechanisms in certain contexts, to generate outcomes, with the outcome of interest in this study being that crossing international borders for planned healthcare. From the outset, we rejected the view of neo-classical economics, and drew on theories of markets being a social systems whereby goods or services are exchanged within a system of social relations and networks [35]. As our review continued, while we still viewed patient travel within a theory of how markets work, we became more aware of the interaction between material and non-material resources, and found the work of Bourdieu resonated with our ongoing synthesis. In this way, as our understanding deepened, the conceptual platform developed, while we still drew on the concepts of markets, we also incorporated aspects of Bourdieu’s work, although this middle range theory was not included in our original protocol [35]. For realist syntheses, the middle range theory is usually an abstracted form of programme theory and tested against corresponding to programme components [20].

Despite not focussing on a particular family of programmes, we were still able to apply the realist logic to our review to develop a theoretical explanation. The use of Bourdieu’s [58–61] work to understand transnational patient travel is also novel. The approach has drawn attention to habitus and capital, and how the pursuit of health is bound up in patients’ everyday practices. As is typical with realist evaluation, while C-M-O configurations have been identified, they are open to further testing and iterative refinement against empirical data [27].

A limitation is that we only included papers and documents in English language and, therefore, may have missed some studies. Limited information was available on the specific socio-economic demographics of patients who cross borders for healthcare, although we were able to identify how access to the different forms of capital facilitated travel choice. While this is a potential limitation of our study, it is consistent with the realist approach which recognises that our understanding of reality is always incomplete [20, 55]. Over time, however, it is possible to contribute to what is understood, and the theories produced in one study may contribute to further cycles of inquiry and theoretical development [20, 55]. Another limitation of our work is that we were not able to fully capture changes in dispositions, or habitus, as a result of cross-border healthcare seeking or undertake a full analysis of the field. Further, our outcome of interest was the decision by patients to cross borders for healthcare. This was not intended to sidestep the important issue of the impact of these decisions on patients themselves, health services in receiving and sending countries, and health financing, including the formal and informal economy that develops around this trade in healthcare and health equity outcomes. There was very limited evidence, however, of how and in what circumstances this cross-border patient movement impacts the health and the continuum of care of those who travel and the broader health systems. There may also be other contextual factors that influence if and how the mechanisms operate, but it is rarely possible to describe every contextual feature. Lastly, this paper did not provide specific recommendations for policy and practice, as is typical of many realist reviews. This was because, unlike other studies using this approach, we did not examine a specific intervention. In this sense, this research is novel.

## Conclusions

This research shows that people from LMICs travel to the same or higher income countries for planned healthcare. Patient movement challenges traditional ways of thinking about public health and the notion of health systems contained within the nation state. Further research is needed to better understand the effects of patient travel and the implications for global health, including legal and regulatory issues, ethical concerns, data collection and transnational infections, and how to harness the benefits of patient travel without exacerbating existing health inequalities.

## Additional file

Additional file 1: Study characteristics. (DOCX 13 kb)

## Abbreviations

CMO: Context, mechanism, outcome
LMIC: Low and middle income country
WHO: World Health Organization
