# Supplementary material for: A realist synthesis of cross-border patient movement from low and middle income countries to similar or higher income countries
Source: Global Health. 2017 Aug 29;13:68. doi: 10.1186/s12992-017-0287-8 (PMC5575883; doi:10.1186/s12992-017-0287-8)
Supplement: Additional file 1: — Study characteristics. (DOCX 13 kb) [file 12992_2017_287_MOESM1_ESM.docx]

*Additional file 1* Study characteristics

| Author, year | Sending country | Receiving country | Study type |
| --- | --- | --- | --- |
| Ahwireng-Obeng, 2011 (66) | Botswana,  Malawi, Ethiopia, Zambia, Mozambique, Zimbabwe, Angola, Namibia, Ghana,  Mauritius, Nigeria, Senegal, Swaziland, Tanzania and Uganda. | South Africa | Mixed-methods |
| Allen, 2013 (67) | Uganda | Kenya | Qualitative |
| Bochaton, 2013 (68) | Lao PDR | Thailand | Mixed-methods |
| Bochaton, 2015 (69) | Lao PDR | Thailand | Mixed-methods |
| Chaleunvong, 2013 (70) | Lao PDR | Thailand | Quantitative |
| Crush, 2015 (15) | African countries | South Africa | Review |
| Guendelman, 1992 (71) | Mexico | United States | Quantitative |
| Inhorn, 2011 (72) | Middle-Eastern countries | Middle-Eastern countries | Review |
| Inhorn, 2010 (73) | India, Lebanon, United Arab Emirates,  Britain, Pakistan, Sudan, Philippines, and Palestine. | United Arab Emirates | Qualitative |
| Inhorn, 2012 (74) | 50 different countries, spanning 6 continents (North America, South America, Australia, Europe, Asia and Africa) | United Arab Emirates and the United States | Qualitative |
| Kangas, 2002 (75) | Yemen | Jordan | Qualitative |
| Kangas, 2007 (76) | Yemen | India and Jordan | Mixed-methods |
| Kangas, 2010 (77) | Yemen | India and Jordan | Qualitative |
| Kangas, 2010 (78) | Yemen | India | Qualitative |
| Kangas, 2011(79) | Yemen | Jordan | Qualitative |
| Lautier, 2008 (80) | Libya | Tunisia | Review |
| Mamun, 2013 (81) | Bangladesh | India, Singapore, Thailand | Quantitative |
| Maung, 2014 (82) | Burma | Thailand | Quantitative |
| Moghimehfar, 2011 (83) | Iraq, Afghanistan,  Pakistan | Iran | Quantitative |
| Ormond, 2015 (84) | Indonesia | Malaysia | Qualitative |
| Ormond, 2014 (11) | Indonesia | Malaysia | Qualitative |
| Smith, 2013 (85) | Indonesia | Malaysia | Qualitative |
| Toyota, 2013 (86) | Indonesia | Malaysia | Qualitative |
| Yeoh, 2013 (87) | Indonesia,  Singapore, other (Japan, China,  Vietnam, Cambodia, Korea, US, Africa, Sweden, Canada, Thailand,  Australia, France and Germany). | Malaysia | Quantitative |
| Yu, 2012 (88) | China, Japan, Korea | Korea | Quantitative |
| Zanini, 2013 (2) | Ecuador | Italy | Qualitative |
| Zhang, 2013 (89) | China | United States, Republic of Korea (Korea), Thailand, Singapore and India. | Quantitative |
| Pocock, 2011 (16) | Indonesia, Cambodia, ASEAN countries | Thailand, Singapore, Vietnam and Malaysia | Review |
| Crush, 2012 (90) | African countries | South Africa | Review |
| Whittaker, 2015 (91) | United Arab Emirates, Oman, Qatar, Kuwait, Ethiopia, Mongolia, the United States, Britain, Australia, Vietnam, Myanmar, Cambodia, Laos, and Macau | Thailand | Qualitative |
| Rahman, 2000 (92) | Bangladesh | India | Quantitative |
